# Established cell surface markers efficiently isolate highly overlapping populations of skeletal muscle satellite cells by fluorescence-activated cell sorting

**DOI:** 10.1186/s13395-016-0106-6

**Published:** 2016-11-08

**Authors:** Claire C. Maesner, Albert E. Almada, Amy J. Wagers

**Affiliations:** 1 Department of Stem Cell and Regenerative Biology, Harvard University and Harvard Stem Cell Institute, Cambridge, MA 02138 USA; 2 Paul F. Glenn Center for the Biology of Aging, Harvard Medical School, Boston, MA 02115 USA

**Keywords:** Satellite cells, Surface marker, Fluorescence-activated cell sorting

## Abstract

**Background:**

Fluorescent-activated cell sorting (FACS) has enabled the direct isolation of highly enriched skeletal muscle stem cell, or satellite cell, populations from postnatal tissue. Several distinct surface marker panels containing different positively selecting surface antigens have been used to distinguish muscle satellite cells from other non-myogenic cell types. Because functional and transcriptional heterogeneity is known to exist within the satellite cell population, a direct comparison of results obtained in different laboratories has been complicated by a lack of clarity as to whether commonly utilized surface marker combinations select for distinct or overlapping subsets of the satellite cell pool. This study therefore sought to evaluate phenotypic and functional overlap among popular satellite cell sorting paradigms.

**Methods:**

Utilizing a transgenic *Pax7*-zsGreen reporter mouse, we compared the overlap between the fluorescent signal of canonical paired homeobox protein 7 (*Pax7*) expressing satellite cells to cells identified by combinations of surface markers previously published for satellite cells isolation. We designed two panels for mouse skeletal muscle analysis, each composed of markers that exclude hematopoietic and stromal cells (CD45, CD11b, Ter119, CD31, and Sca1), combined with previously published antibody clones recognizing surface markers present on satellite cells (β1-integrin/CXCR4, α7-integrin/CD34, and Vcam1). Cell populations were comparatively analyzed by flow cytometry and FACS sorted for functional assessment of myogenic activity.

**Results:**

Consistent with prior reports, each of the commonly used surface marker schemes evaluated here identified a highly enriched satellite cell population, with 89–90 % positivity for Pax7 expression based on zsGreen fluorescence. Distinct surface marker panels were also equivalent in their ability to identify the majority of the satellite cell pool, with 90–93 % of all Pax7-zsGreen positive cells marked by each of the surface marker schemes. The direct comparison among surface marker schemes validated their selection for highly overlapping subsets of cells. Functional analysis in vitro showed no differences in the abilities of cells sorted by these different methods to grow in culture and differentiate.

**Conclusions:**

This study demonstrates the equivalency of several previously published and widely utilized surface marker schemes for isolating a highly purified and myogenically active population of satellite cells from the mouse skeletal muscle, which should facilitate cross-comparison of data across laboratories.

**Electronic supplementary material:**

The online version of this article (doi:10.1186/s13395-016-0106-6) contains supplementary material, which is available to authorized users.

## Background

The skeletal muscle is a highly dynamic tissue with a remarkable capacity for rapid regeneration following injury. The essential precursors driving this regenerative process are the satellite cells—a group of mononuclear, self-renewing, and tissue-resident adult stem cells comprising 2–5 % of all muscle nuclei [3, 8, 31]. Satellite cells are classically defined by their stereotypical location between the sarcolemma and basal lamina of multinucleated myofibers and by their expression of the canonical satellite cell regulatory gene, paired homeobox protein 7 (*Pax7*) [25, 30, 34]. Upon muscle damage and myofiber membrane disruption, satellite cells are exposed to extracellular cues that activate them for re-entry into cell cycle via induced expression of key myogenic regulatory transcription factors [2, 12, 13, 16]. Satellite cells then proliferate and divide [1, 17] to generate differentiated myoblasts that facilitate repair, as well as self-renewed satellite cells that replenish the muscle stem cell pool [8, 12, 23].

Recent studies have emphasized molecular and functional heterogeneity within the satellite cell pool [6, 11, 26]. For example, satellite cells expressing higher levels of *Pax7* have been shown to display lower metabolic activity, proliferate less, and possess an increased propensity to self-renew [28]. These transcriptional and functional differences have prompted researchers to classify muscle progenitors in the satellite cell pool hierarchically, with the hope of identifying the best candidate population for clinical and pre-clinical research. Yet, such studies remain dependent on robust methods for collecting these primary cells for study.

Fluorescent-activated cell sorting (FACS) using specific cell surface marker combinations is widely employed as a robust and reliable method for isolating mouse satellite cells from freshly harvested muscle-associated mononuclear cells. The use of cell surface markers has the advantage that it is broadly applicable across a range of mouse strains, ages, and genotypes. Congruently, populations lacking myogenic capabilities have been excluded using other surface markers, such as Sca1 and CD45, which mark muscle-resident and muscle-infiltrating hematopoietic and fibroadipogenic cell types [3, 22]. Yet, within the non-hematopoietic, non-fibroadipogenic subset of muscle mononuclear cells, many surface marker schemes have been reported to positively enrich satellite cells. Some of the cell surface antigens employed are used independently of other positive markers, including VCam1, α7-integrin, NCam1, cMet, m-Cadherin, and Synd3/4 [5, 15, 18, 21, 24, 34], and some are used in combination, including β1-integrin and CXCR4 or α7-integrin and CD34 [11, 14, 19, 29, 32, 33, 35]. However, it remains unknown if all of these surface proteins are expressed on the same satellite cells. Given the known heterogeneity in the satellite cell pool, this creates difficulty for drawing conclusions about satellite cell biology across studies employing different sorting paradigms.

In this study, we used a transgenic *Pax7*-zsGreen reporter mouse [7] and a panel of several commonly used surface markers to compare methods for identifying adult mouse satellite cells within a myofiber-associated cell pool by FACS. The markers examined—β1-integrin and CXCR4 [32], α7-integrin and CD34 [29], and VCam1 [18, 24]—were chosen based on the prevalence of their use in the field and the availability of well-characterized monoclonal antibodies with compatible fluorochromes. This approach allowed us to explore the overlap in expression of each surface marker-defined population, the efficiency with which each enables selection of muscle satellite cells, and whether multiple surface markers are indeed expressed on the same satellite cells.

## Methods

### Mice and antibodies

*Pax7*-zsGreen reporter mice [7] were bred and maintained on a C57BL/6J background at the Harvard University Biological Research Institute. Adult animals (6–12 weeks) of both sexes were used along with age-matched C57BL/6J controls.

The following antibodies were used at a 1:200 titer to exclude cells within hematopoietic and stromal cell types from further analysis: allophycocyanin (APC)-conjugated anti-mouse Ly6A/E (Sca1: Biolegend—clone E3-161.7), APC anti-CD31 (Biolegend—clone 390), APC anti-CD45 (Biolegend—clone 30-F11), APC anti-CD11b (Mac1: Biolegend—clone M1/70), APC anti-Ter119 (Biolegend—clone Ter119). All satellite cell-enriching antibodies were rigorously titered under standard staining conditions of 1E6 cells per 100 μL volume to optimize the separation between positive and negative staining cell populations (Additional file 1: Figure S1). The optimal dilutions for each antibody under these conditions were APC-Cy7 anti-CD29 (β1-integrin, titer 1:100: Biolegend—clone HMβ1-1), biotin anti-CD184 (CXCR4, titer 1:100: BD Biosciences—clone 2B11), phycoerythrin (PE) anti-CD106 (VCam1, titer 1:100: Lifetech—clone M/K-2), PE anti-α7-integrin (titer 1:200: AbLab—clone R2F2), Alexa-Fluor700 anti-CD34 (titer 1:25, e-Biosciences—clone Ram34). A PE-Cy7-streptavidin conjugate (titer 1:200) was used for biotin detection of CXCR4 staining. Both propidium iodide (PI; titer 1:1000: Sigma) and calcein blue (titer 1:1000: Lifetech) were added 5–10 min preceding flow cytometry to evaluate cell viability.

### Satellite cell analysis and isolation

Myofiber-associated (MFA) cells were prepared for analysis and FACS from intact skeletal muscles using a two-step enzymatic digestion protocol previously described [14, 32]. Muscles included in this preparation were the triceps brachii, latissimus dorsi, pectoralis, extensor digitorum longus, gastrocnemius, quadriceps, soleus, tibialis anterior, and abdominis. Briefly, pooled muscles harvested from *Pax7*-zsGreen mice were digested with 0.2 % collagenase type II (285 U/mg, Lifetech) in Dulbecco’s modified Eagle medium (DMEM; Lifetech) for 90 min at 37 °C. The enzyme was inactivated with 20 % fetal bovine serum (FBS) in F10, and muscles were washed in phosphate buffered saline (PBS) and triturated to mechanically dissociate individual fibers from the tissue. The collected fibers were gravitationally sedimented through a series of settling steps at 37 °C for 25, 15, and 10 min, respectively. The fibers were next digested with 0.0125 % collagenase type II and 0.05 % dispase (1.81 U/mg, Lifetech) in F10 for 30 min at 37 °C to release mononuclear cells from fibers. Cells were further separated by pipette, spun at 400 rpm for 15 s to pellet debris, and the supernatant was filtered through a 70 μm cell strainer. Subsequently, cells were counted using a hemocytometer, brought to a total staining volume of 1E6 cells per 100 μL of staining media (2 % FBS in Hank’s Balanced Salt Solution) and split for staining. Based on commercial availability of particular fluorophore conjugates for each of the specific antibody clones needed for this study, we combined satellite cell markers in the following two combinations: β1-integrin, CXCR4, and VCam1 or β1-integrin, CXCR4, α7-integrin, and CD34. All cells were stained with antibodies recognizing Sca1, CD31, CD45, Mac1, and Ter119. Primary antibody incubations were performed on ice for 35 min and streptavidin incubations for 20 min. All cells were sorted twice to maximize cell purity; the three groups of cells sorted from the myogenic fractions were [PI^−^, calcein^+^, Sca1^−^, CD31^−^, CD45^−^, Mac1^−^, Ter119^−^, β1-integrin^+^, CXCR4^+^], [PI^−^, calcein^+^, Sca1^−^, CD31^−^, CD45^−^, Mac1^−^, Ter119^−^, VCam1^+^], or [PI^−^, calcein^+^, Sca1^−^, CD31^−^, CD45^−^, Mac1^−^, Ter119^−^, α7-integrin^+^, CD34^+^]. For both sorting and analysis, all gates were established using fluorescence minus one (FMO) controls [27], which contained cells stained with all fluorophores except the one conjugated to the surface marker of interest. Cell sorting was performed at the Harvard Stem Cell Institute Flow Cytometry core, and flow cytometry data were analyzed using FlowJo (BD Biosciences, 2015) analysis software.

### Myogenic colony formation, myogenic commitment, and differentiation assays

For clonal analyses, cells were sorted at one cell per well into 96-well plates containing myogenic growth medium (F10, 20 % Donor Horse Serum, 1 % penicilin-streptomyocin, 1 % glutamax). Sorted cells were cultured for 5 days, with daily addition of 5 ng/mL basic fibroblast growth factor, at which point wells were assessed for visible cell growth. Wells were scored as “positive” in this assay (i.e., containing a cell colony) if two or more cells were detected. Unscored wells contained either no visible cells or only one cell.

For analysis of myogenic commitment, freshly isolated satellite cells were expanded in 24-well plates containing growth media with daily addition of basic fibroblastic growth factor for 5 days. After 5 days, cells were harvested from plates using a final concentration of 2.5 μM EDTA pH 8.0 for 15 min, counted, and seeded at a density of 3000 cells per well in growth medium in 96-well plates. Twenty-four hours later, immunofluorescence was performed using either anti-Pax7 (DSHB, 1/15), anti-MyoD (1/20, sc-760, clone M-318), or anti-Myogenin (1/50, BD Biosciences-556358) primary antibodies.

For analysis of differentiation, cells were expanded for 5 days in 24-well plates containing myogenic growth medium with daily addition of basic fibroblastic growth factor. After 5 days, cells were harvested, re-plated at a density of 3000 or 8000 cells per well and switched to myogenic differentiation media (F10, 2 % Donor Horse Serum, 1 % penicilin-streptomyocin, 1 % glutamax). Cells were allowed to differentiate for 72 h and then fixed with 4 % paraformaldehyde and stained for the differentiation marker myosin heavy chain (MyHC; Sigma fast-M4276 and slow-M8421). Hoechst 33342 was added to mark nuclei.

For all assays, 24- and 96-well plates were coated with a final concentration of 1 μg/ml of collagen type I (Sigma, #C7661) and 10 μg/mL of laminin (Invitrogen, #23017-015) 24 h prior to seeding cells.

### Cell imaging and quantification

Imaging and quantification were performed using a Celigo imaging cytometer and software (Nexcelom, 2012). A “differentiation index” was defined using developer software and in-house scripts to determine the fraction of total nuclei contained within myosin heavy chain+ myotubes. Multi-nucleated myotubes at times contain highly dense nuclei that can be difficult to correctly segment into individual nuclei using imaging software. This circumstance can lead to the classification as a single object of a cluster that contains more than one nucleus. Thus, the use of count-based quantification in such instances can lead to inaccurate quantification. To overcome this limitation, we used integrative intensity of nuclear stain (DAPI or Hoechst) for each object rather than raw counts. To ensure capture of fused MyHC+ myotubes and not mononuclear cells expressing MyHC, we first defined the mean area (112.27 μm^2^) and standard deviation (104.35 μm^2^) of all single nuclei (objects) from myogenic cultures (representing six individual wells from each cell population: β1-integrin^+^CXCR4^+^, Vcam1^+^, or α7-integrin^+^CD34^+^) grown in growth media. Using the equation below, we defined myotubes as any object having an area greater than the mean mononuclear cell area +1 standard deviation (216.62 μm^2^) and positive for MyHC. Positivity for MyHC was determined based on secondary only control staining. The differentiation index was then defined as the sum of the integrated intensity of Hoechst within all MyHC+ objects meeting the aforementioned size criteria (nuclei within myotubes) divided by the sum of the integrated intensity of ALL objects (total nuclei in mononucleated and multinucleated cells) per well. We find this index to largely recapitulate results obtained from calculation of fusion index using manual cell/nuclear counting (data not shown).

### Immunofluorescent staining and microscopy

Cells were fixed with 4 % paraformaldehyde for 20 min and then permeabilized with 0.3 % Triton-X for 20 min at room temperature. Cells were washed for 5 min with PBST three times after fixing and permeabilization. Cells were incubated in blocking media (PBS-T, 3 % bovine serum albumin, 5 % normal goat serum, 8 % protein concentrate (vector labs), and 0.2 % Tween-20) for 2 h at room temperature. Cells were incubated with primary antibody solution in blocking media with antibodies overnight at 4 °C at the following dilutions: 1:200 anti-fast MyHC (Sigma M4276) and 1:100 anti-slow MyHC (Sigma M8421), anti-Pax7 (DSHB, 1/15), anti-MyoD (1/20, sc-760, clone M-318), and anti-Myogenin (1/50, BD Biosciences-556358). After thorough washing (three times, 15 min each in PBST), the secondary antibody solution containing 1:250 goat anti-mouse 488 (Lifetech) or 1:250 goat anti-rabbit 488 (Lifetech) and Hoechst 33342 (Thermofisher) in blocking buffer was incubated for 1 h at room temperature (dilutions used for all immunofluorescent antibodies had previously been titrated in C2C12 differentiated myotubes). Images of sorted cells were obtained using an Axio-Observer inverted light microscope and AxioVision software (Zeiss, 2015). A merged multidimensional acquisition was set up using a 365-nm reflector for Hoechst and a 470-nm reflector for MyHC, Pax7, MyoD, and MyoG.

### Statistics

All statistical analyses were performed using Prism 6 software (GraphPad Software Inc., 2015). Group analyses were done using ordinary one-way ANOVA and comparison between two groups using Student’s *t* test.

## Results

**Fig. 1:**
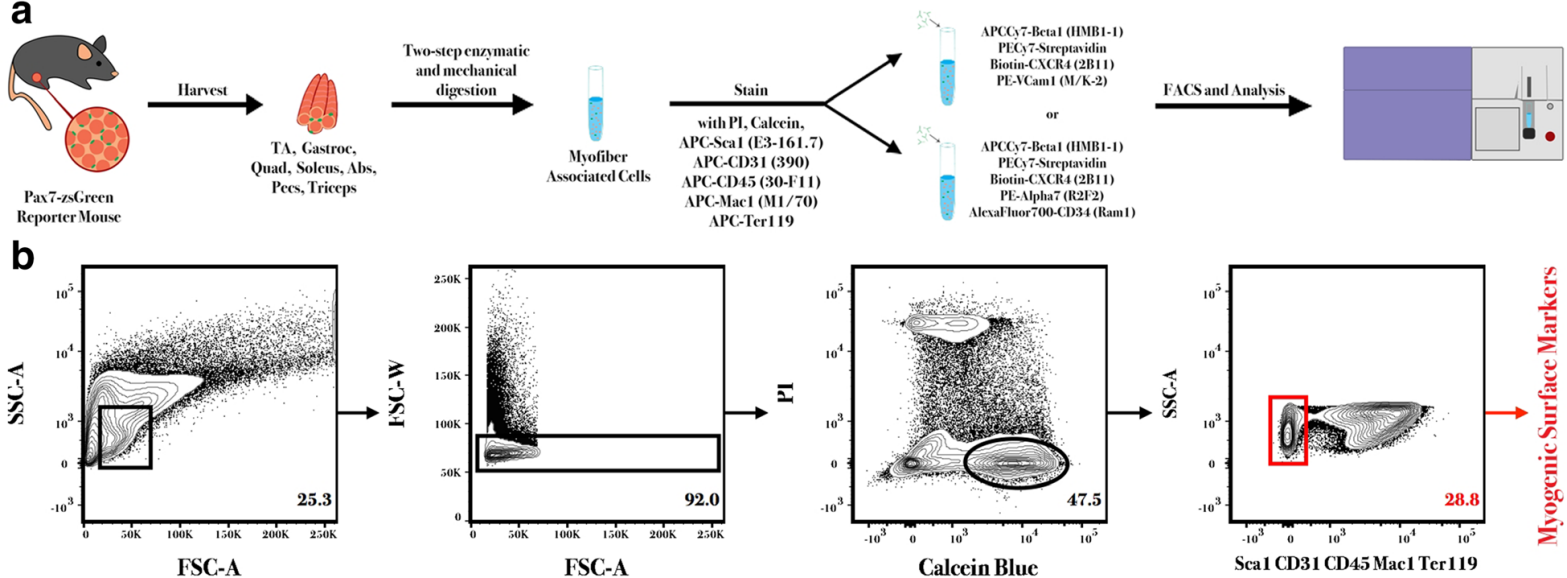
Experimental design and applied gating strategy for flow cytometry*.* **a** Skeletal muscles were harvested from Pax7-zsGreen animals and mononuclear and myofiber-associated cells were purified using a two-step enzymatic and mechanical digestion procedure. All cells were stained with the viability markers PI and calcein, as well as negatively selecting markers. Due to fluorophore overlap, positive selecting antibodies were split between two staining conditions including all hematopoietic and stromal factors (Sca1, CD31, CD45, Mac1, and Ter119) and either β1-integrin, CXCR4, and VCam1 or β1-integrin, CXCR4, α7-integrin, and CD34. Cells were analyzed and sorted by flow cytometry. **b** Gating conditions applied to all downstream analysis included initial physical parameter gate to exclude cells larger than our target population, viability selection of cells that were PI^−^ and calcein^+^, and exclusion of hematopoietic and stromal cell types. All comparative flow cytometric analyses (see Figs. 2, 3, and 4) began with this Sca1, CD31, CD45, Mac1, and Ter119 negative cell population (*red*)

We sought to investigate the co-expression of *Pax7* and the surface markers β1-integrin, CXCR4, VCam1, α7-integrin, and CD34 in freshly isolated myofiber-associated cell populations from adult mouse skeletal muscle. To accomplish this, we harvested fresh muscle tissue from *Pax7*-ZsGreen transgenic mice [7], isolated myofiber-associated mononuclear cells using a two-step protocol of enzymatic digestion and mechanical trituration [14, 32], and performed flow cytometric analysis using negative and positive selection markers (Fig. 1a).

Within our panel, we created a combinatorial exclusion of hematopoietic and stromal cell surface markers within the APC channel, using anti-mouse Sca1, CD31, CD45, Mac1, and Ter119 (Table 1). Positive-selecting surface markers were chosen from antibody clones previously characterized for satellite cell enrichment [5, 29, 32] and titrated under standard staining conditions prior to use (Additional file 1: Figure S1). Optimal titers were identified from the dilution that yielded the largest separation between positively and negatively staining cell populations without increasing background signal relative to an unstained control. Titration curves were considered together with histograms of fluorescent intensity to arrive at the chosen dilutions of 1:200 for α7-integrin, 1:100 for β1-integrin, CXCR4, and VCam1, and 1:25 for CD34 (Additional file 1: Figure S1). Due to the limited availability of fluorophores directly conjugated to the clonal antibodies used, myofiber-associated cells collected from each *Pax7*-zsGreen animal were split into two staining conditions containing antibodies recognizing either VCam1 or α7-integrin and CD34 surface markers in tandem with β1-integrin and CXCR4 (Table 1). Our gating analysis prior to positive-selection remained constant throughout all analyses (Fig. 1b) and included stringent physical parameters to increase resolution within the myogenic fraction (Additional file 2: Figure S2A), as well as a viability selection and an exclusion of hematopoietic and stromal cell types.

**Table 1:** Surface markers used within flow cytometry panels

Marker		Clone	Fluorophore conjugate	Titer
Ly6A/E	Sca1	E3-161.7	APC	1:200
CD31		390	APC	1:200
CD45		30-F11	APC	1:200
CD11b	Mac1	M1/70	APC	1:200
	Ter119	Ter119	APC	1:200
CD29	β1-integrin	HMβ1-1	APC-Cy7	1:100
CD184	CXCR4	2B11	PE-CY7	1:100
CD106	Vcam	M/K-2	PE	1:100
	α7-integrin	R2F2	PE	1:200
CD34		Ram1	AlexaFluor700	1:25

Cluster of differentiation (CD) number, where relevant, target antigen, clone name, fluorophore conjugate, and optimal titer are listed for each antibody used in this study

**Fig. 2:**
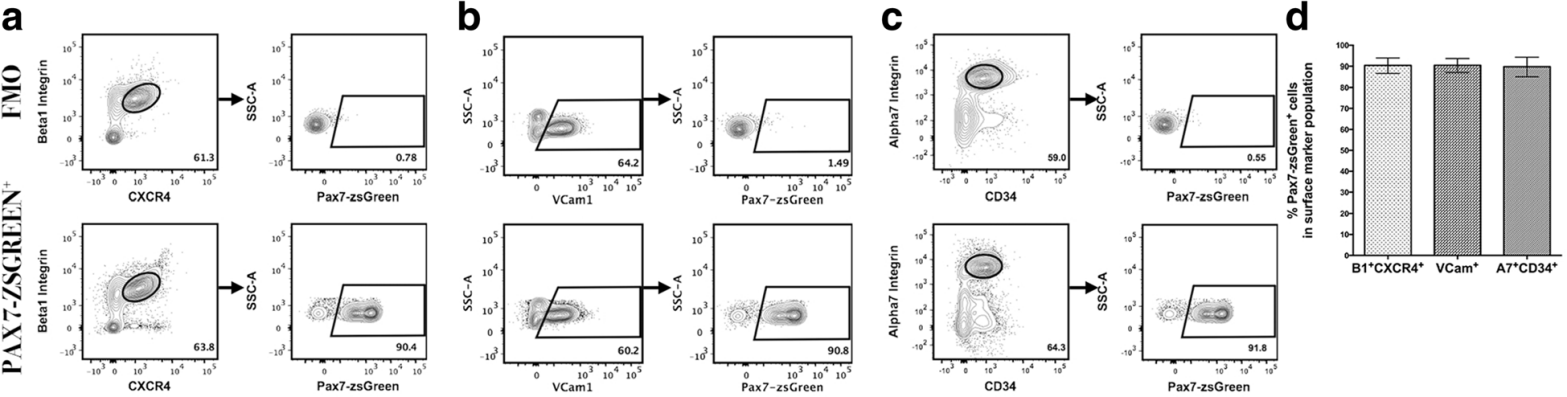
The majority of skeletal muscle satellite cells isolated by distinct surface marker combinations express *Pax7*. All gating conditions were set using non-transgenic C57BL/6J mouse myofiber-associated cells to distinguish cells with positive signal for Pax7-zsGreen (see “FMO” control, **a**–**c**, *top row*). **a**–**c** The populations marked by β1-integrin and CXCR4, VCam1, or α7-integrin and CD34 are highly enriched for cells expressing Pax7-zsGreen. **d** Comparative analysis across surface marker strategies shows no significant differences in Pax7-zsGreen expression between groups (*n* = 12)

We first investigated the frequency of *Pax7-zsGreen* ^+^ cells within each surface marker defined fraction in young adult mice (aged 6–12 weeks). No expression of zsGreen was seen within the fluorescence minus one (FMO) controls (cells isolated from wild-type C57BL/6J mice), allowing for clear definition of expressing cells within all subsequent samples (Fig. 2a–c). The large majority of cells in the β1-integrin^+^CXCR4^+^ (90.3 ± 3.59 %, *n* = 12), Vcam1+ (90.41 ± 3.22 %, *n* = 12), and α7-integrin^+^CD34^+^ (89.71 ± 4.55 %, *n* = 12) fractions were positive for Pax7-expression (Fig. 2a–c). No significant differences were apparent in the frequencies of *Pax7*-expressing cells in the cell populations identified using each surface marker combination (*p* = 0.8883, Fig. 2d). In addition, selection for α7-integrin alone was equivalently effective for enriching Pax7+ cells as the combination of α7-integrin and CD34 (Additional file 3: Figure S3A). These data confirm prior reports [9, 10, 21, 24, 29] that each of these surface marker paradigms isolates a population highly enriched for Pax7-expressing satellite cells.

**Fig. 3:**
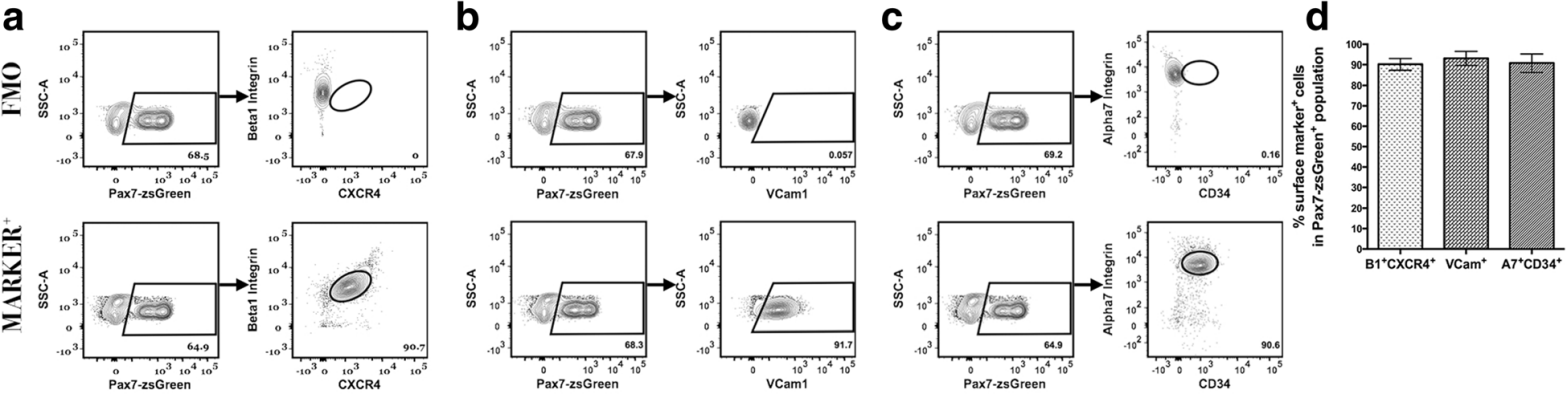
Canonical *Pax7*-expressing satellite cells are positive for distinct surface marker combinations. All gating conditions were set using Pax7-zsGreen mouse cells stained as FMO controls to distinguish cells with positive signal of the marker population (**a**–**c**, *top row*). **a**–**c** Satellite cells expressing Pax7-zsGreen are highly marked by β1-integrin and CXCR4, VCam1, or α7-integrin and CD34 markers. **d** Comparative analysis shows no significant differences in surface marker composition among Pax7^+^ satellite cells (*n* = 12)

We also examined the surface marker composition of the identified fraction of *Pax7-zsGreen* ^+^ cells. Using FMO controls, we first defined our satellite cell population and found that the majority of *Pax7-zsGreen* ^+^ cells stained positively for β1-integrin and CXCR4 (90.24 ± 2.774 %, *n* = 12), VCam1 (93.07 ± 3.473 %, *n* = 12), and α7-integrin and CD34 (90.78 ± 4.454 %, *n* = 12) (Fig. 3a–c). No significant differences were seen in comparing the percentages of surface marker positive cells among these *Pax7-zsGreen* ^+^ cells (*p* = 0.1610, Fig. 3d). Interestingly, the analysis of the percent surface marker positive cells within the Pax7+ population differed slightly (but significantly) for α7 alone versus α7+CD34^+^ (double positive) cells (Additional file 3 Figure S3B), suggesting that selection with both α7-integrin and CD34 may isolate a slightly more restricted subset of satellite cells than selection with α7-integrin alone, possibly by specifically enriching “quiescent” satellite cells, as reported previously [4]. While we did note a discrete separation of high and low *Pax7* expression within the *Pax7-zsGreen* ^+^ population, no correlation was seen between the level of *Pax7* expression and the level of expression of any particular surface marker (Additional file 4 Figure S4). Given these results, we conclude that all of these surface markers are present on a majority proportion of *Pax7*-expressing satellite cells. Our results further indicate that multiple surface markers are likely co-expressed by individual *Pax7* ^+^ cells.

**Fig. 4:**
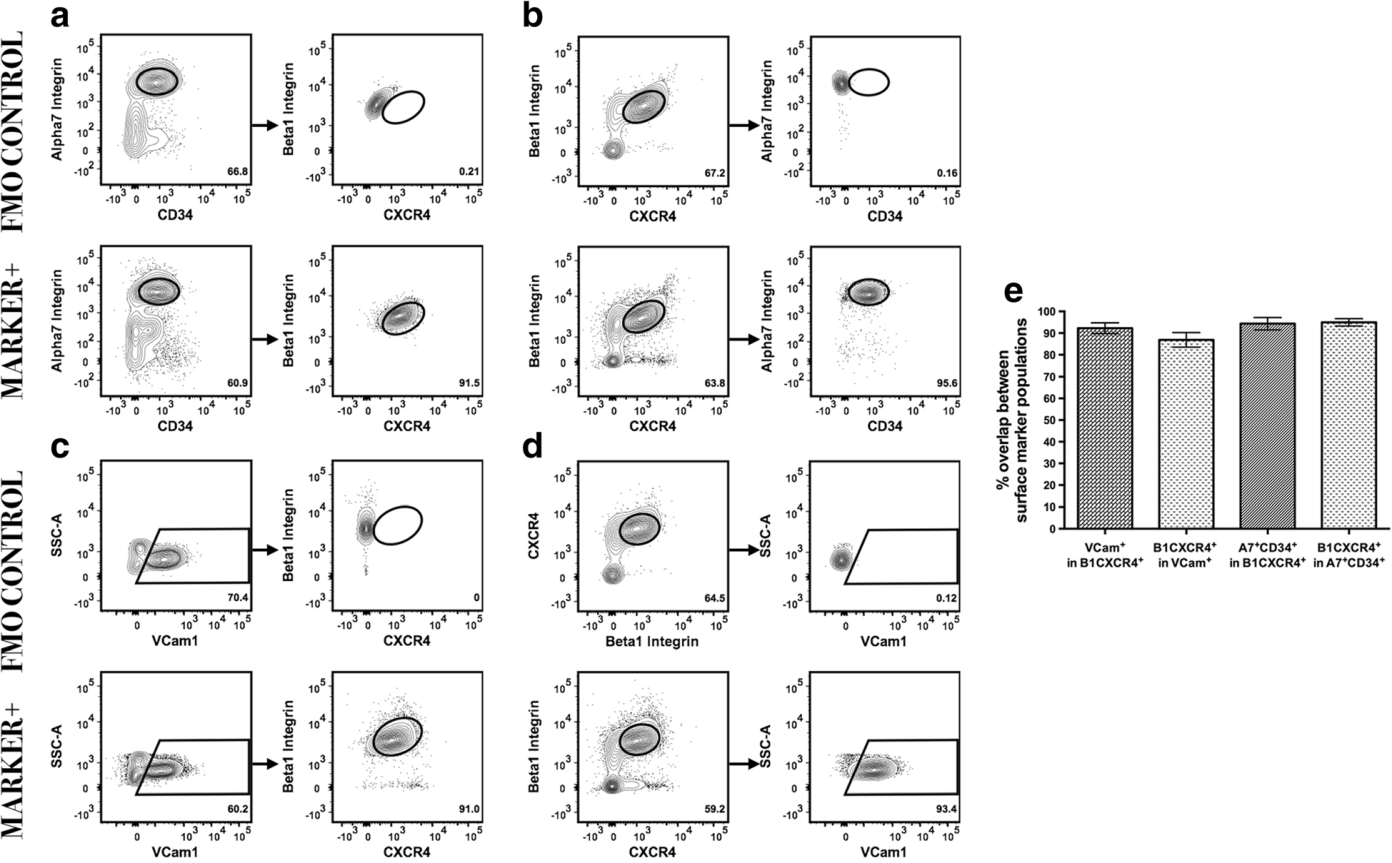
High overlap between unique surface marker combinations that enrich for skeletal muscle satellite cells. All gating conditions were set using cells stained as FMO controls to distinguish cells with positive signal of the marker population (**a**–**d**, *top row*). **a** Cells expressing β1-integrin and CXCR4 markers also express α7-integrin and CD34. **b** Cells expressing α7-integrin and CD34 also express β1-integrin and CXCR4. **c** Cells expressing VCam1 also express β1-integrin and CXCR4. **d** Cells expressing β1-integrin and CXCR4 markers also express VCam1. **e** Comparative analysis of surface marker presence within surface marker defined populations indicates expression of multiple surface markers on the same satellite cells (*n* = 12)

To further characterize surface marker expression within the satellite cell pool, we again used FMO controls to identify the positive surface marker populations. Of the α7-integrin^+^ and CD34^+^ cells within the myogenic fraction, the majority (94.95 ± 1.683 %, *n* = 12) were positive for both β1-integrin and CXCR4 (Fig. 4a). Similarly, within the fraction of cells positive for VCam1, the majority (86.88 ± 3.34 %, *n* = 12) were positive for both β1-integrin and CXCR4 (Fig. 4c). Most cells that were positive for β1-integrin and CXCR4 were positive for VCam1 (92.26 ± 2.488 %, *n* = 12) and for α7-integrin and CD34 (94.34 ± 2.86 %, *n* = 12) (Fig. 4b, d). Although direct comparison of the populations stained by VCam1 or α7-integrin and CD34 was not possible within the parameters of this study, the high overlap of both populations with β1-integrin- and CXCR4-stained cells demonstrates their homology. Statistical analysis showed some variation within the groups, but this comparison may be conceptually unimportant given the robustness of each scheme for labeling cells expressing the canonical satellite cell marker *Pax7*. While expanding our initial selection gate decreased the relational percentage and increased experimental variation between biological replicates, the high overlap among the distinct sorting schemes was still well represented across all comparison groups (Additional file 2: Figure S2B–F).

**Fig. 5:**
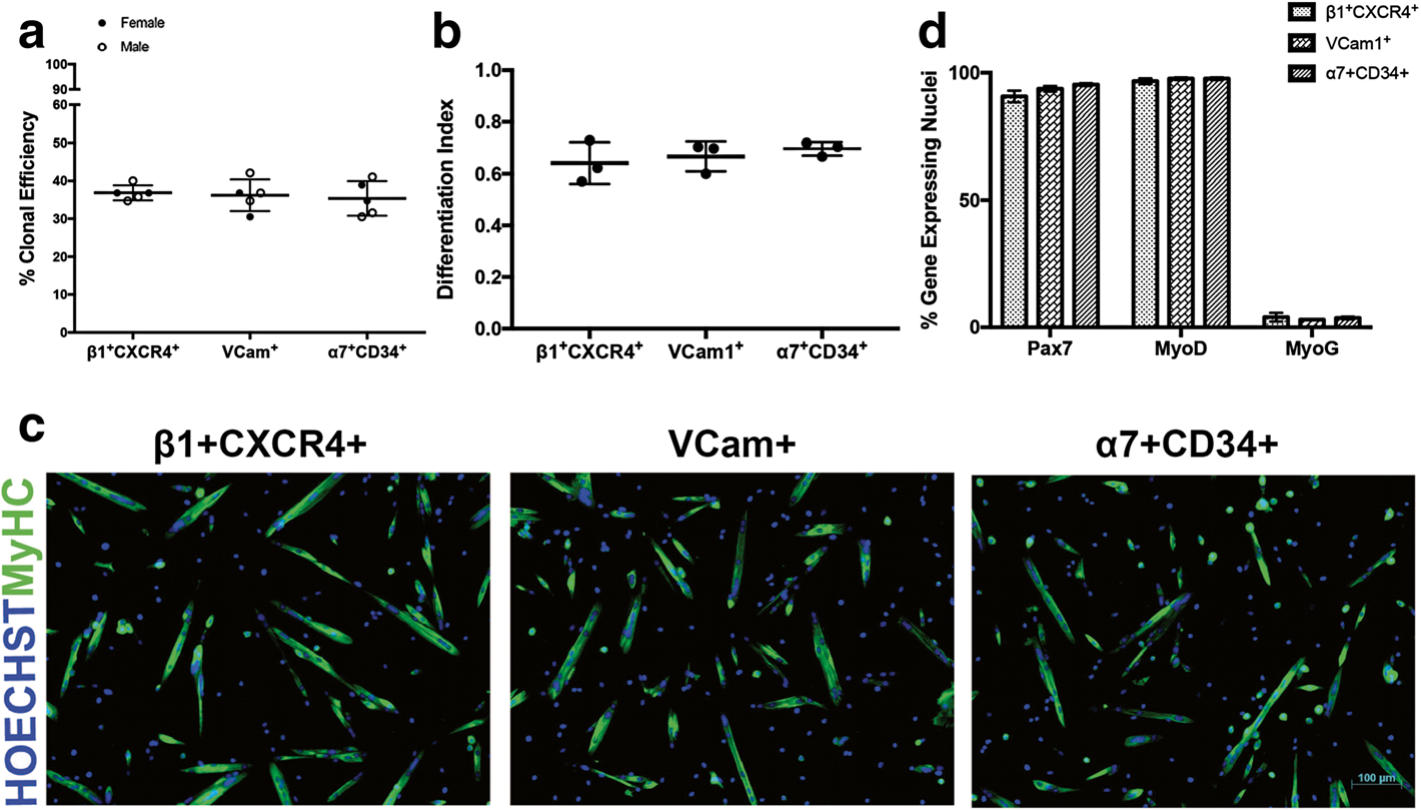
Functional characterization of skeletal muscle progenitor cells isolated by distinct surface marker combinations. **a** Similar levels of colony formation were seen across cells isolated by β1-integrin and CXCR4, VCam1, and α7-integrin and CD34 sorting paradigms. Data were collected for cells harvested independently from *n* = 5 mice (two female and three male). Each *dot* represents colony-forming efficiency of an individual mouse, calculated from analysis of at least 95 wells. *Overlay* represents mean ± SD. **b** No differences in myogenic differentiation indices (see “Methods” section) among β1-integrin and CXCR4, VCam1, and α7-integrin and CD34 sorted populations. Data were collected for cells harvested independently from *n* = 3 female mice. Each *dot* represents one mouse, with two technical replicates per biological replicate. *Overlay* indicates mean ± SD. **c** Representative **×**10 images of cultures quantified in (**b**), derived from sorted β1-integrin and CXCR4 (*left*), VCam1 (*middle*), and α7-integrin and CD34 (*right*) cell populations after 72 h in differentiation media. **d** Quantification of the percentage of Pax7, MyoD, or MyoG protein expressing cells within myogenic cultures expanded in growth media for 5 days. *Error bars* represent standard deviations. *N* = 3, with two technical replicates per biological replicate

To further validate the equivalence of satellite cells isolated by the surface marker schemes investigated above, we pursued functional assays to compare the in vitro clonogenic and myogenic potentials of sorted cells. When plated at a single-cell density to assess the proportion of cells that are capable of surviving and expanding under culture conditions supportive of myogenic cell proliferation, cells isolated by β1-integrin^+^CXCR4^+^, VCam1^+^, and by α7-integrin^+^CD34^+^ formed colonies with equal efficiencies (36.84 ± 1.968 %, 36.21 ± 4.184 %, 35.37 ± 4.562 %, respectively; *p* = 0.8263; Fig. 5a, *n* = 5). To gain insight into early events in myogenic commitment of satellite cells isolated by each strategy, we cultured freshly sorted cells for 5 days in myogenic growth media followed by immunostaining for Pax7, MyoD, and MyoG protein. These results indicate that each cell population generated cultures of similar cell density containing a comparable percentage of Pax7+, MyoD+, and MyoG+ cells (Fig. 5d and Additional file 5: Figure S5). Similarly, the analysis of myogenic differentiation capabilities among these sorted cell populations showed comparable myogenic activity, as assessed visually (Fig. 5c and Additional file 5: Figure S5 and Additional file 6: Figure S6) and by a quantitative differentiation index that considers the ratio of nuclei incorporated into MyHC-positive cells relative to the total nuclei per well (64.09 ± 8.08 %, 66.74 ± 5.78 %, 69.68 ± 2.65 %; *p* = 0.466; Fig. 5b). Altogether, these comparisons indicate that the three satellite cell surface marker schemes analyzed here detect largely overlapping cell populations and can be used interchangeably to select for muscle satellite cells in young adult mice.

## Discussion

Using surface markers to distinguish satellite cells has been integral for primary cell isolation by FACS from mouse skeletal muscle. This approach has greatly advanced our understanding of satellite cell biology, with studies examining all facets of satellite cell fate and function, including assessments of gene expression profile and muscle regenerative capacity. However, a lack of clarity regarding the relationship among the different surface marker paradigms used to identify satellite cells has hindered cross-study comparison in the field. Our rigorous comparison of satellite cell surface marker expression, Pax7 positivity, and myogenic activity clearly demonstrates that three highly utilized surface marker combinations efficiently isolate a highly enriched and largely overlapping population of cells from the adult mouse skeletal muscle.

Of note, in our analysis of satellite cells from Pax7-zsGreen transgenic mice, we did not observe enriched expression of any of the surface markers evaluated within the Pax7^hi^ or Pax7^lo^ satellite cell subsets (see Additional file 4: Figure S4). Our observations contrast with a prior analysis of Pax7-nGFP transgenic mice [28], which indicated that the top 10 % of nGFP+ cells expressed significantly higher levels of CD34 (by FACS and RT-PCR) and of CXCR4 (by RT-PCR). The discrepancy in these two studies regarding the correlation between Pax7 and cell surface marker expression can likely be attributed to one or more key differences in the animal models used to track Pax7 expression. In particular, different spectral properties, sub-cellular localization, and protein stability of monomeric, nuclear GFP protein versus cytoplasmically localized, tetrameric ZsGreen could affect the ability to resolve highly similar sub-populations by FACS. In addition, differences in transgene integration site could impact the overall expression levels of the reporter genes in the two strains in a potentially cell type specific or cell subset specific manner. In any event, regardless of the underlying reasons, these data suggest that the Pax7-zsGreen mouse line may not be optimal for separating Pax7^hi^ cells with unique functional characteristics as described by [28].

Also of importance, while our data strongly suggest that the three different sorting strategies examined here are essentially interchangeable, both phenotypically and functionally, for isolating satellite cells from the uninjured muscles of adult mice, and thus directly comparable across studies, they do not address the equivalency of these sorting schemes in older animals or from the previously injured muscle. Thus, it will be important in future studies to rigorously examine how expression of these surface markers in satellite cells may be influenced by various muscle injury protocols [20] or in aged or diseased environments [7]. Nonetheless, these results provide a first step towards harmonizing the field with respect to satellite cell identification and analysis, and we hope these data provide a useful benchmark to facilitate the exchange of information across diverse laboratory groups studying muscle satellite cell biology.

## Conclusions

- Satellite cell populations freshly isolated from mouse skeletal muscle by surface marker panels using Sca1, CD31, CD45, Mac1, and Ter119 exclusion with positive enrichment for either β1-integrin and CXCR4, VCam1, or α7-integrin and CD34 are highly overlapping.
- Equivalent, highly enriched populations of satellite cells can be isolated by each of these three distinct selection schemes.
- These three surface marker schemes are phenotypically and functionally interchangeable

## Additional files

Additional file 1: Figure S1. Titration of positive selecting surface markers. Overlay of histograms of fluorophore intensity (top) and resulting titration curves (bottom, plotted as mean fluorescence intensity (MFI) of the positively staining cell population achieved for each antibody dilution (range 1:25–1:200) from all surface marker antibodies used are shown to demonstrate how antibody titers giving the largest separation between positive and negative populations were chosen. Within the histograms, each dilution is identified by color: red, FMO control; blue, 1:25; orange 1:50; light green 1:100; dark green, 1:200. Cells were stained with fluorophores from all utilized channels, and titrations were performed within the PI^−^, calcein+, Sca1^−^, CD31^−^, CD45^−^, Mac1^−^, and Ter119^−^ population.
Additional file 2: Figure S2. Alternative gating strategy shows same trends in population overlap*.* A) Back-gating analysis supporting the use of a restrictive FSC/SSC gate for satellite cell identification. Plots shown for two representative Pax7-zsGreen transgenic mice. Less than 5 % of selected cells fall outside the restrictive scatter gate. B) Gating strategy includes all previously used parameters with more inclusive initial physical parameter selection (compare to SSC vs. FSC gate in Fig. 1b). C–F) Analysis of β1-integrin and CXCR4 compared to either VCam1 or α7-integrin and CD34 expressing cells shows similarly high levels of surface marker identification. For each marker combination, FMO controls are shown in the top row and marker stained cells in the bottom row.
Additional file 3: Figure S3. Comparative analysis of satellite cells identified by expression of α7-integrin alone or as α7-integrin^+^CD34^+^. A) Gating scheme for identification of Pax7+ cells among α7-integrin^+^ or α7-integrin^+^CD34^+^ cells and quantification of the percent Pax7+ cells within each population. The populations marked by α7-integrin alone and by α7-integrin and CD34 are equivalently highly enriched for cells expressing Pax7-zsGreen (*n* = 12 mice/group). B) Gating scheme and quantification of the percent α7-integrin^+^ or α7-integrin^+^CD34^+^ cells among Pax7-zsGreen+ cells. The combination of α7-integrin^+^ and CD34^+^ identified a slightly smaller subset of Pax7-zsGreen+ cells, as compared to α7-integrin^+^ alone (*n* = 12 mice per group). **p* < 0.05 by Student’s *t* test.
Additional file 4: Figure S4. Correlational data for expression of each surface marker and Pax7 expression level. Cells segregated by different levels of Pax7-expression show equivalent levels of expression of CXCR4, β1-integrin, α7-integrin, CD34, and VCam1. Marker identity indicated below each histogram/contour plot. A) Gating scheme for total Pax7+ subset. B) Gating of Pax7^hi^ and Pax7^lo^ populations based on apparent separation in total Pax7+ cell histogram (grey histogram at left, gated as in A). Red curve represents high Pax7 expressors, and blue curve represents low Pax7 expressors. C) Gating of top 10 % Pax7^hi^ and bottom 10 % Pax7^lo^ populations based on the parameters defined by [28]. Red contour plots represent high Pax7 expressors, and blue contour plots represent low Pax7 expressors.
Additional file 5: Figure S5. Antibody validation for Pax7, MyoD, and MyoG in growth and differentiation media. Representative images for Pax7- (top), MyoD- (middle), and myogenin (bottom)-positive staining along with DAPI (cell nuclei) from β1-integrin^+^CXCR4^+^ satellite cell progeny cultured in growth media (left side) or in differentiation media for 72 h (right side). All images within a group (Pax7, MyoD, MyoG) were set to the same exposure at which no signal was observed in the secondary only control. Consistent with anticipated results, Pax7 is detected in the majority of cells in growth media, but declines appreciably in myogenic cultures in differentiation media. Similarly, MyoG is expressed in few cells in growth media, but is present in many cells after switching to differentiation media. MyoD is expressed by a majority of cells in both conditions.
Additional file 6: Figure S6. Equivalent myogenic differentiation of sorted satellite cell populations. Representative ×3.5 (top) and ×20 (bottom) images of myogenic cultures seeded from 8000 cells, derived from sorted β1-integrin and CXCR4 (left), VCam1 (middle), and α7-integrin and CD34 (right) cell populations after 72 h in differentiation media.

## Abbreviations

FACS: Fluorescence-activated cell sorting
FBS: Fetal bovine serum
FMO: Fluorescence minus one
MFA: Myofiber associated
MyHC: Myosin heavy chain
PBS(-T): Phosphate-buffered saline (-Tween)
PE: Phycoerythrin
SMP: Skeletal muscle precursor
