# Supplementary figures and images for: Established cell surface markers efficiently isolate highly overlapping populations of skeletal muscle satellite cells by fluorescence-activated cell sorting
Source: Skelet Muscle. 2016 Nov 8;6:35. doi: 10.1186/s13395-016-0106-6 (PMC5100091; doi:10.1186/s13395-016-0106-6)

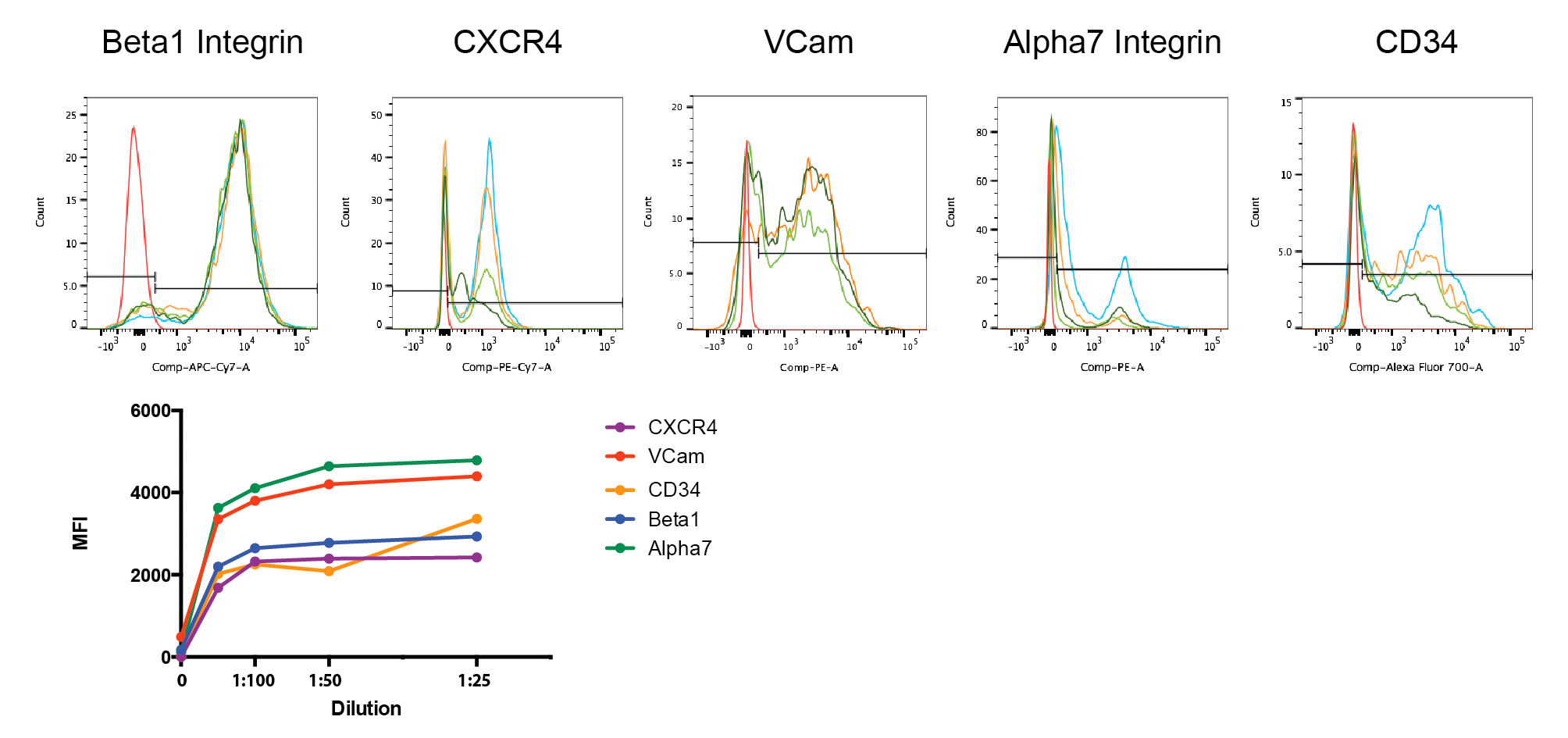

Supplement: Additional file 1: Figure S1. — Titration of positive selecting surface markers. Overlay of histograms of fluorophore intensity (top) and resulting titration curves (bottom, plotted as mean fluorescence intensity (MFI) of the positively staining cell population achieved for each antibody dilution (range 1:25–1:200) from all surface marker antibodies used are shown to demonstrate how antibody titers giving the largest separation between positive and negative populations were chosen. Within the histograms, each dilution is identified by color: red, FMO control; blue, 1:25; orange 1:50; light green 1:100; dark green, 1:200. Cells were stained with fluorophores from all utilized channels, and titrations were performed within the PI−, calcein+, Sca1−, CD31−, CD45−, Mac1−, and Ter119− population. [file 13395_2016_106_MOESM1_ESM.tif]

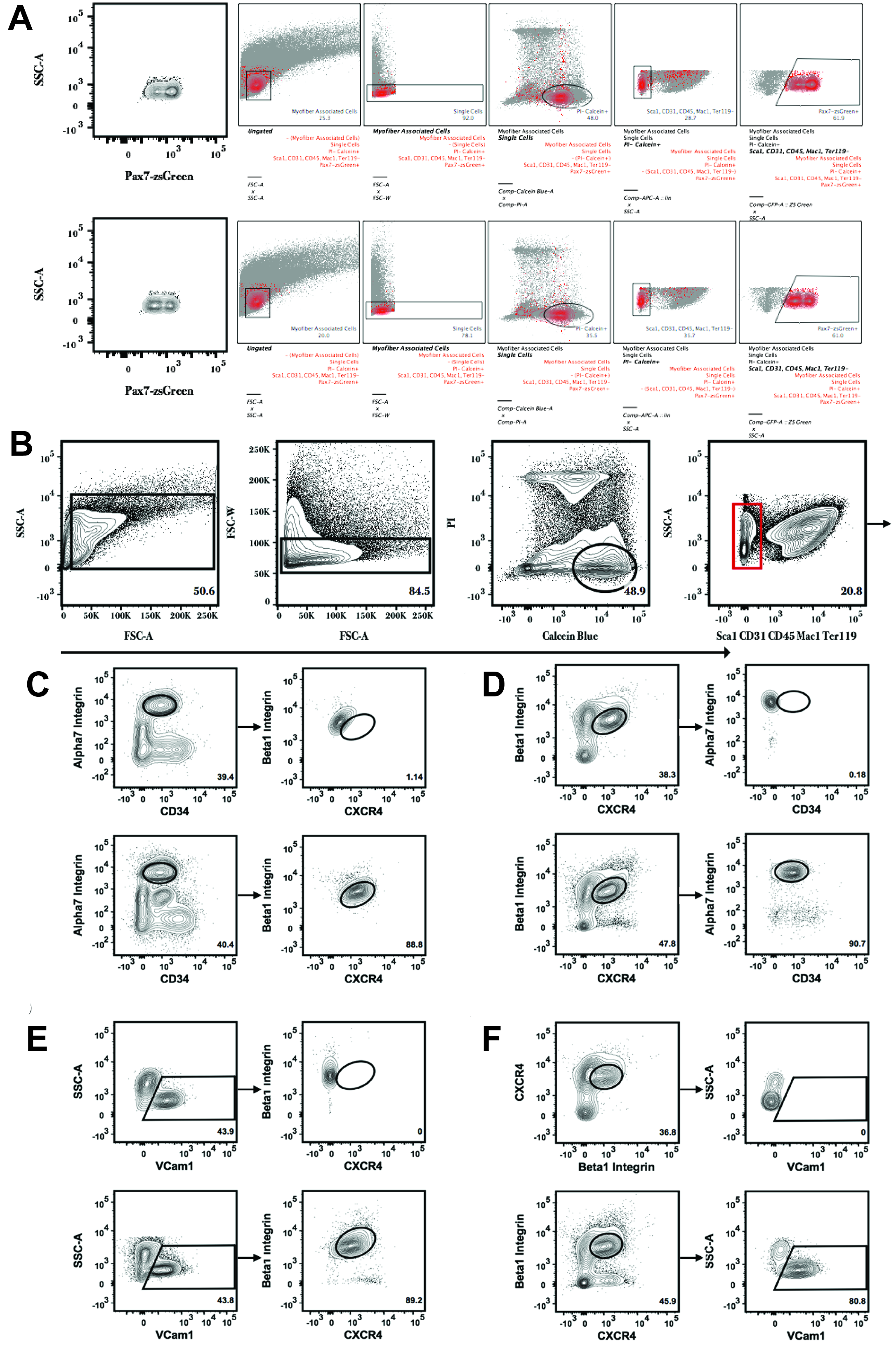

Supplement: Additional file 2: Figure S2. — Alternative gating strategy shows same trends in population overlap. A) Back-gating analysis supporting the use of a restrictive FSC/SSC gate for satellite cell identification. Plots shown for two representative Pax7-zsGreen transgenic mice. Less than 5 % of selected cells fall outside the restrictive scatter gate. B) Gating strategy includes all previously used parameters with more inclusive initial physical parameter selection (compare to SSC vs. FSC gate in Fig. 1b). C–F) Analysis of β1-integrin and CXCR4 compared to either VCam1 or α7-integrin and CD34 expressing cells shows similarly high levels of surface marker identification. For each marker combination, FMO controls are shown in the top row and marker stained cells in the bottom row. [file 13395_2016_106_MOESM2_ESM.tif]

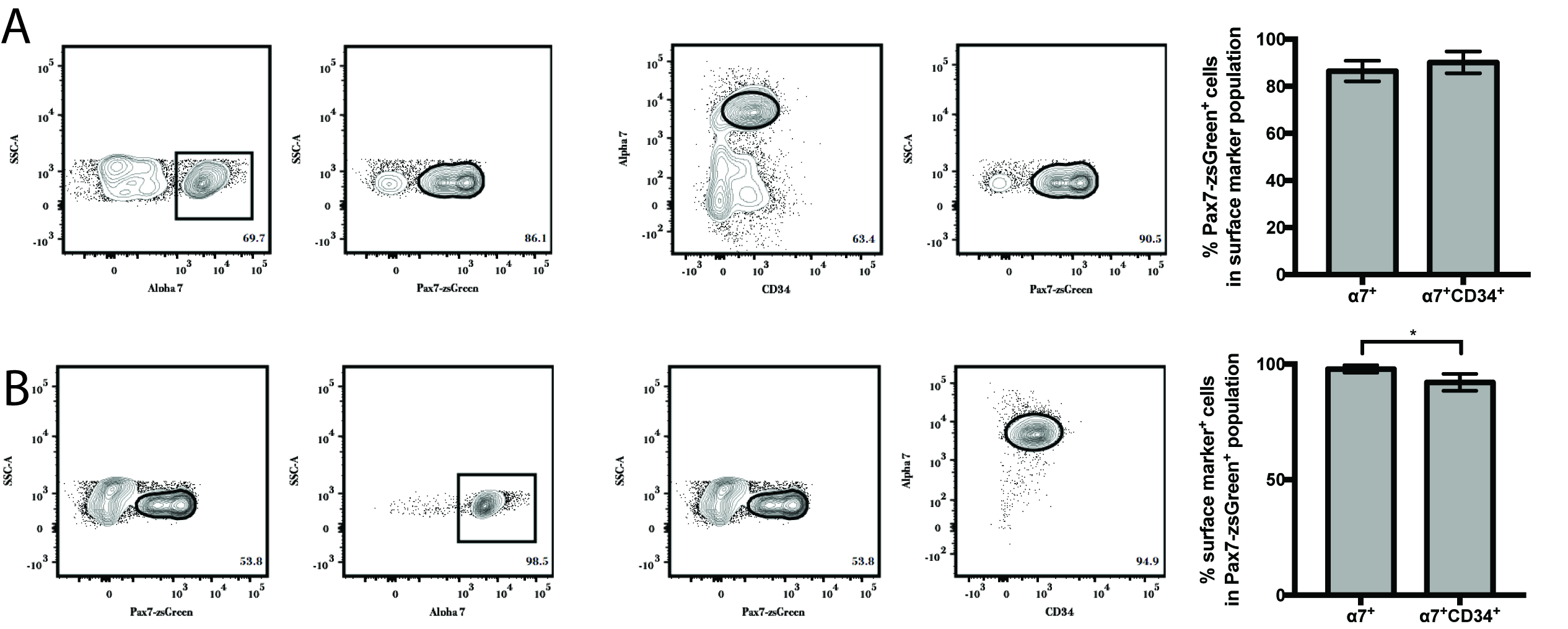

Supplement: Additional file 3: Figure S3. — Comparative analysis of satellite cells identified by expression of α7-integrin alone or as α7-integrin+CD34+. A) Gating scheme for identification of Pax7+ cells among α7-integrin+ or α7-integrin+CD34+ cells and quantification of the percent Pax7+ cells within each population. The populations marked by α7-integrin alone and by α7-integrin and CD34 are equivalently highly enriched for cells expressing Pax7-zsGreen (n = 12 mice/group). B) Gating scheme and quantification of the percent α7-integrin+ or α7-integrin+CD34+ cells among Pax7-zsGreen+ cells. The combination of α7-integrin+ and CD34+ identified a slightly smaller subset of Pax7-zsGreen+ cells, as compared to α7-integrin+ alone (n = 12 mice per group). *p < 0.05 by Student’s t test. [file 13395_2016_106_MOESM3_ESM.tif]

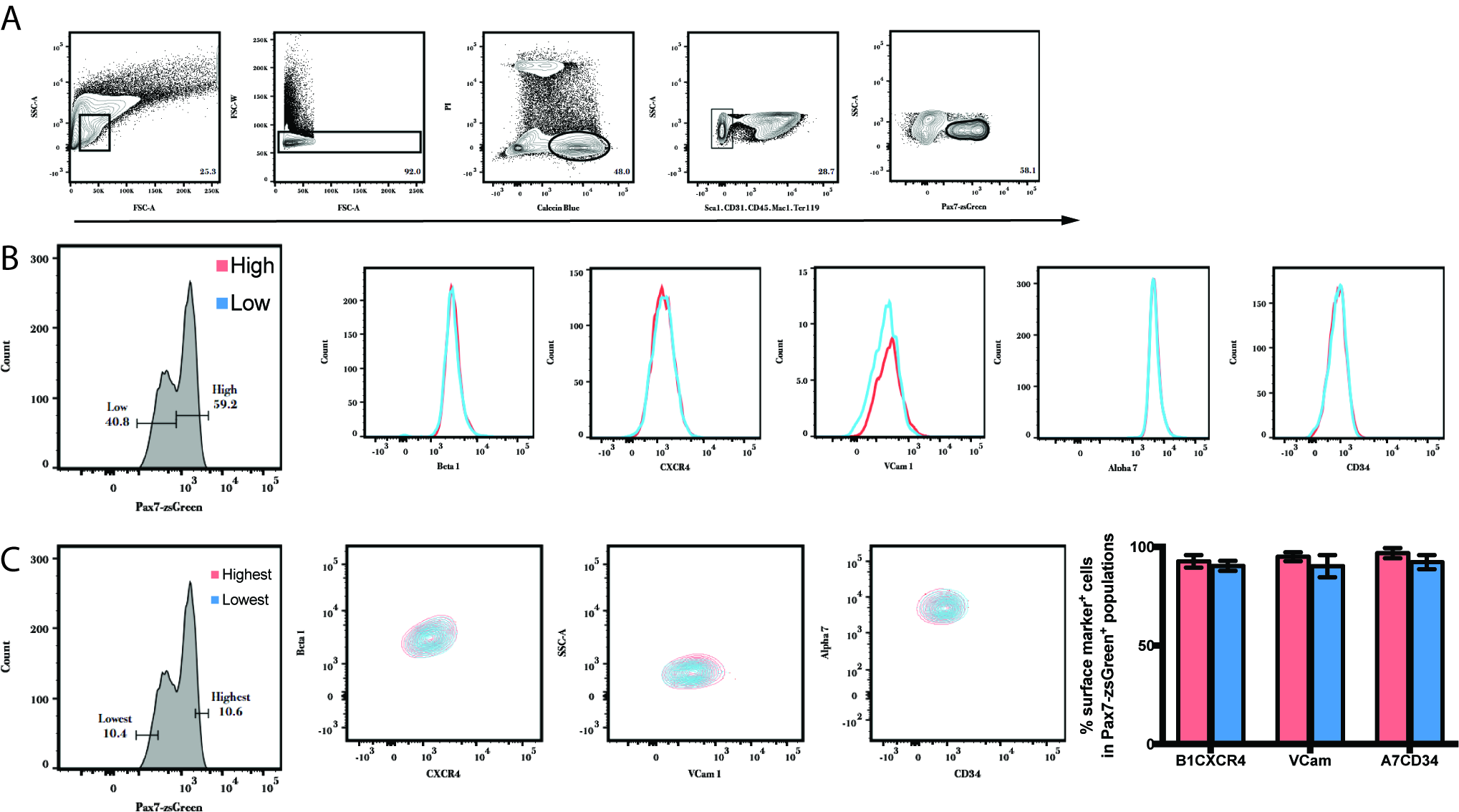

Supplement: Additional file 4: Figure S4. — Correlational data for expression of each surface marker and Pax7 expression level. Cells segregated by different levels of Pax7-expression show equivalent levels of expression of CXCR4, β1-integrin, α7-integrin, CD34, and VCam1. Marker identity indicated below each histogram/contour plot. A) Gating scheme for total Pax7+ subset. B) Gating of Pax7hi and Pax7lo populations based on apparent separation in total Pax7+ cell histogram (grey histogram at left, gated as in A). Red curve represents high Pax7 expressors, and blue curve represents low Pax7 expressors. C) Gating of top 10 % Pax7hi and bottom 10 % Pax7lo populations based on the parameters defined by [28]. Red contour plots represent high Pax7 expressors, and blue contour plots represent low Pax7 expressors. [file 13395_2016_106_MOESM4_ESM.tif]

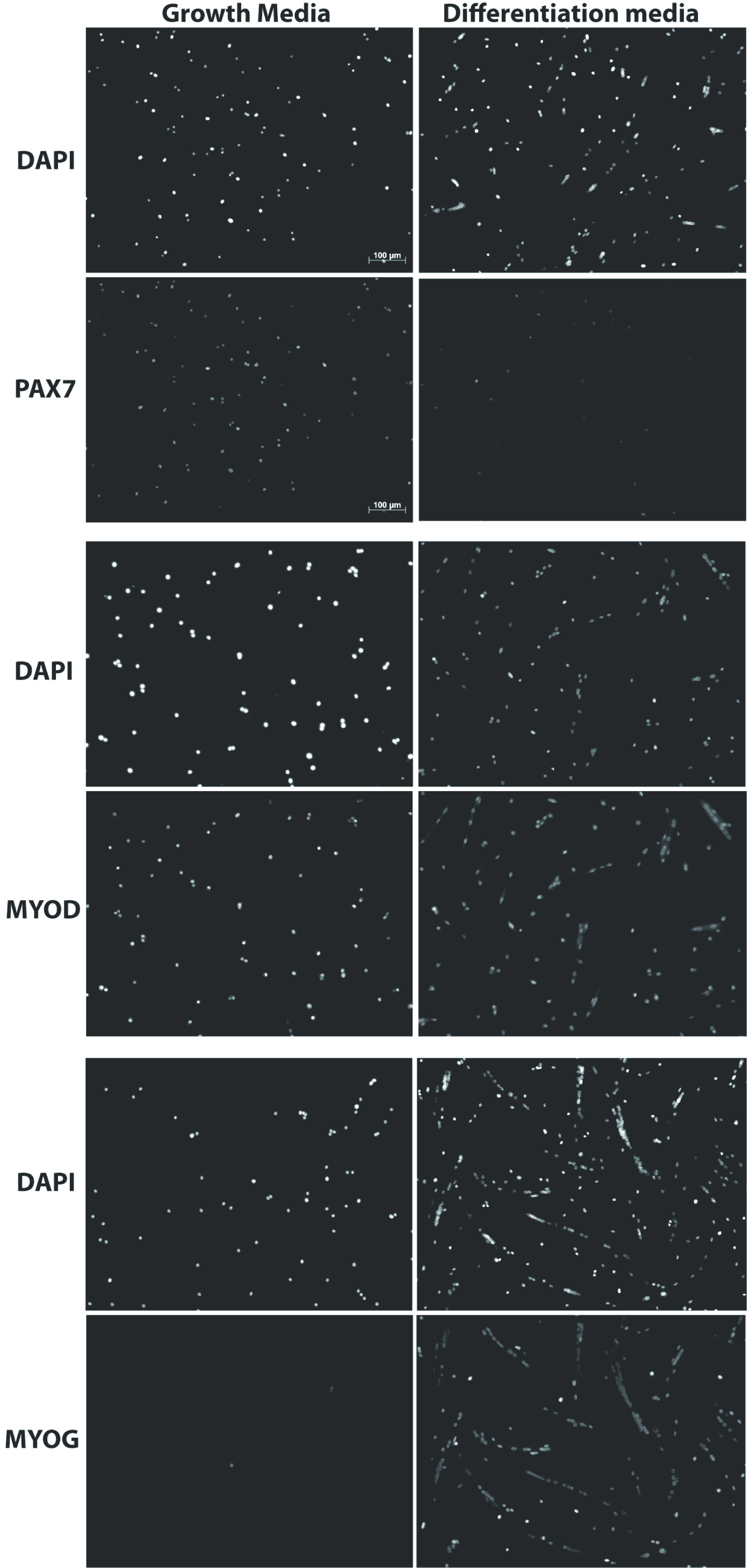

Supplement: Additional file 5: Figure S5. — Antibody validation for Pax7, MyoD, and MyoG in growth and differentiation media. Representative images for Pax7- (top), MyoD- (middle), and myogenin (bottom)-positive staining along with DAPI (cell nuclei) from β1-integrin+CXCR4+ satellite cell progeny cultured in growth media (left side) or in differentiation media for 72 h (right side). All images within a group (Pax7, MyoD, MyoG) were set to the same exposure at which no signal was observed in the secondary only control. Consistent with anticipated results, Pax7 is detected in the majority of cells in growth media, but declines appreciably in myogenic cultures in differentiation media. Similarly, MyoG is expressed in few cells in growth media, but is present in many cells after switching to differentiation media. MyoD is expressed by a majority of cells in both conditions. [file 13395_2016_106_MOESM5_ESM.tif]

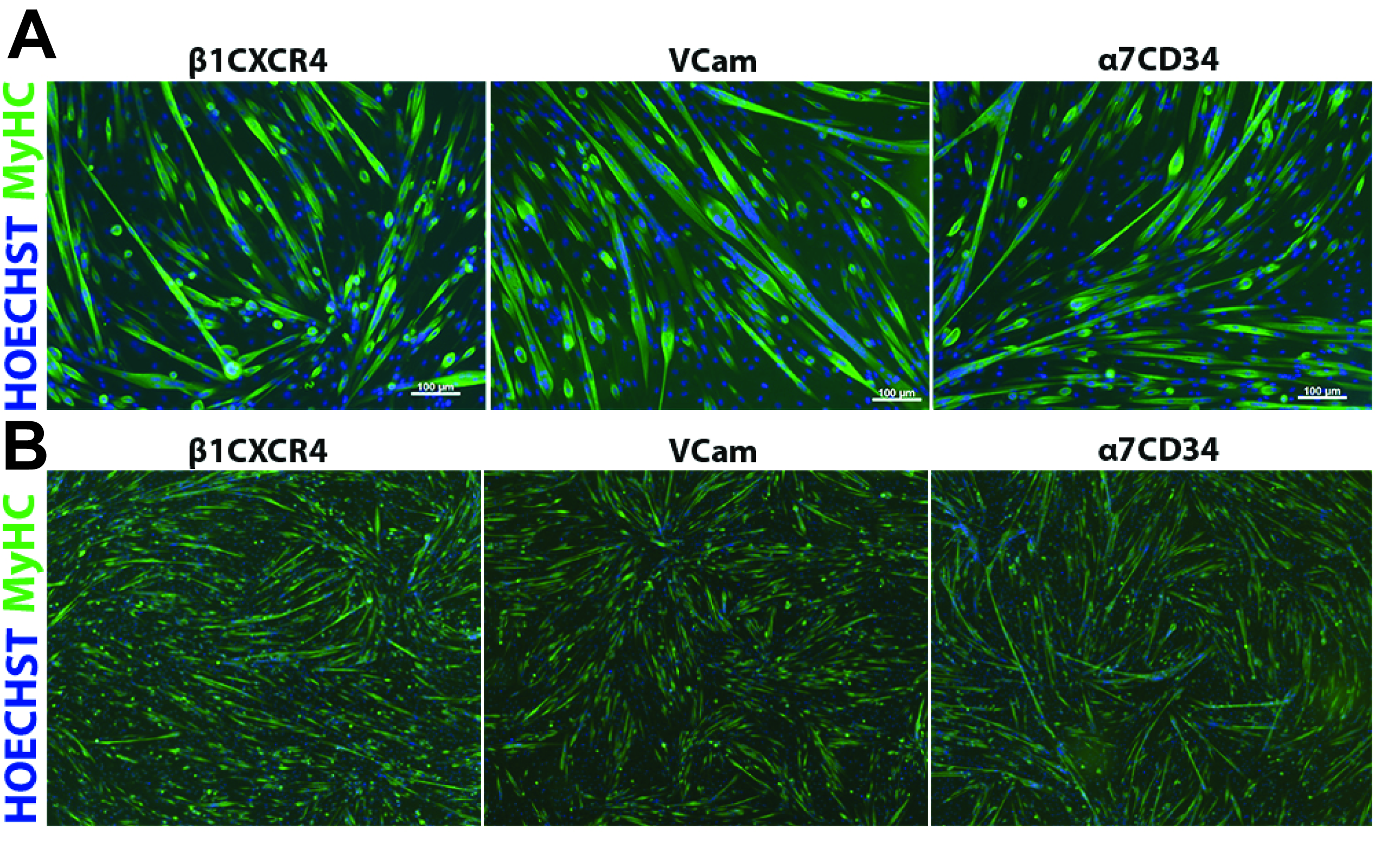

Supplement: Additional file 6: Figure S6. — Equivalent myogenic differentiation of sorted satellite cell populations. Representative ×3.5 (top) and ×20 (bottom) images of myogenic cultures seeded from 8000 cells, derived from sorted β1-integrin and CXCR4 (left), VCam1 (middle), and α7-integrin and CD34 (right) cell populations after 72 h in differentiation media. [file 13395_2016_106_MOESM6_ESM.tif]
